# Band-Selection of a Portal LED-Induced Autofluorescence Multispectral Imager to Improve Oral Cancer Detection

**DOI:** 10.3390/s21093219

**Published:** 2021-05-06

**Authors:** Yung-Jhe Yan, Nai-Lun Cheng, Chia-Ing Jan, Ming-Hsui Tsai, Jin-Chern Chiou, Mang Ou-Yang

**Affiliations:** 1 Institute of Electrical and Control Engineering, National Yang Ming Chiao Tung University, 1001 University Road, Hsinchu City 30010, Taiwan; jerryyan.eed02g@nctu.edu.tw (Y.-J.Y.); chiou@mail.nctu.edu.tw (J.-C.C.); 2 Institute of Biomedical Engineering, National Yang Ming Chiao Tung University, 1001 University Road, Hsinchu City 30010, Taiwan; sky051223@gmail.com; 3 Department of Pathology, China Medical University, 91 Hsueh-Shih Road, Taichung City 40402, Taiwan; cjan1206@mail.cmu.edu.tw; 4 Department of Otolaryngology, China Medical University Hospital, 2 Yuh-Der Road, Taichung City 40447, Taiwan; minghsui@mail.cmuh.org.tw

**Keywords:** AI-based band selection, rule-based band selection, oral squamous cell carcinoma, LED induced autofluorescence, multispectral imager

## Abstract

This aim of this study was to find effective spectral bands for the early detection of oral cancer. The spectral images in different bands were acquired using a self-made portable light-emitting diode (LED)-induced autofluorescence multispectral imager equipped with 365 and 405 nm excitation LEDs, emission filters with center wavelengths of 470, 505, 525, 532, 550, 595, 632, 635, and 695 nm, and a color image sensor. The spectral images of 218 healthy points in 62 healthy participants and 218 tumor points in 62 patients were collected in the ex vivo trials at China Medical University Hospital. These ex vivo trials were similar to in vivo because the spectral images of anatomical specimens were immediately acquired after the on-site tumor resection. The spectral images associated with red, blue, and green filters correlated with and without nine emission filters were quantized by four computing method, including summated intensity, the highest number of the intensity level, entropy, and fractional dimension. The combination of four computing methods, two excitation light sources with two intensities, and 30 spectral bands in three experiments formed 264 classifiers. The quantized data in each classifier was divided into two groups: one was the training group optimizing the threshold of the quantized data, and the other was validating group tested under this optimized threshold. The sensitivity, specificity, and accuracy of each classifier were derived from these tests. To identify the influential spectral bands based on the area under the region and the testing results, a single-layer network learning process was used. This was compared to conventional rules-based approaches to show its superior and faster performance. Consequently, four emission filters with the center wavelengths of 470, 505, 532, and 550 nm were selected by an AI-based method and verified using a rule-based approach. The sensitivities of six classifiers using these emission filters were more significant than 90%. The average sensitivity of these was about 96.15%, the average specificity was approximately 69.55%, and the average accuracy was about 82.85%.

## 1. Introduction

Oral cancer has become a severe health problem in many developing and developed countries. In addition to the economic burden of patients and their families, related medical care has been a central issue of national health. According to the World Health Organization (WHO), 657,000 new cases of oral cancer are diagnosed each year, and more than 330,000 deaths occur due to oral cancer [1]. In Taiwan, oral cancer is ranked as the fifth leading cause of death among common cancers. About 7000 new cases and 3000 deaths of oral cancer occur in Taiwan each year [2]. The incidence rate and mortality rate in Taiwan ranked first and second, respectively, compared with 35 other countries in the OECD. Patients suffering from oral cancer normally have habits of smoking and/or betel-nut chewing in Taiwan and Southeast Asia [3,4,5].

Fluorophore, which is the intermediate product of heme biosynthesis, generates fluorescence after it absorbs a specific excitation light; this phenomenon is called autofluorescence. The fluorophores in human tissue include flavins adenine dinucleotide (FAD), nicotinamide adenine dinucleotide (NADH), and structural protein of collagen, elastin, and keratin. FAD has autofluorescence in the green spectrum between 510 and 570 nm; NADH has autofluorescence in the blue spectrum between 450 and 490 nm; and protoporphyrin has autofluorescence in the red spectrum between 620 and 640 nm [6]. The concentrations of NADH and FAD in tumor tissue were smaller than that of normal tissue because tumor tissue might have higher aerobic metal metabolic activity than normal tissue [7]. This might result in lower intensity of blue and green autofluorescence in tumor tissue compared to that of normal tissue. Structural proteins are considered to make a smaller contribution to fluorescent emission in tumor tissue compared to that of normal tissue because the thickness of the epithelial layer in premalignant and malignant tissues can be larger than that of normal tissue [8,9]. Protoporphyrin may vary with the carcinoma progression; however, it also varies with the number of bacteria on the mucosa [10].

Optical spectroscopy has customarily been used to determine the specified bands for identifying abnormal tissue. Gillenwater et al. [11] found that the fluorescence intensity of abnormal oral tissue significantly decreased in the blue spectrum between 455 and 490 nm compared to that of normal oral tissue. Betz et al. [12] indicated that the fluorescence intensity of malignant mucosa significantly decreased in the green region at 540 and 575 nm compared to that of normal mucosa. Müller et al. [13] demonstrated that the autofluorescent change of NADH and collagen has potential for cancer diagnosis. Majumder et al. [14] measured the spectrum of autofluorescence in squamous cell carcinoma excited by a nitrogen laser, and the accuracy of classifying the carcinoma from the normal squamous cell was over 85% based on total principal component regression. Schwarz et al. reported that the fluorescent spectrum of different depths in oral tissue could enhance the detection of optical changes associated with premalignant because the collagen in the underlying stroma might change with the progression of malignant tumor tissue [15]. Mallia et al. [16] indicated that the ratio of fluorescent intensity at 500 nm to that at 645, 705, and 685 nm could discriminate hyperplasia from dysplastic and normal tissues. In addition to optical spectroscopy, hyperspectral imaging systems (HIS) can measure not only the spatial distribution of the reflectance or transmittance but also the spectral distribution in the specified spectrum range. The hyperspectral imaging system can measure over 100 bands of the spectra. In recent decades, the system has been used in many types of research, including remote sensing, food production, medical detection, and agriculture applications. In a previous study, we developed the embedded relay lens microscopic hyperspectral imaging system (ERL-MHSI) and employed the system in cancer-related research [17]. Depending on the spatial resolution and spectral resolution of the ERL-MHSI system, the data can be analyzed with hyperspectral morphological images. This is a useful tool for research but may not be appropriate for the quick screening of oral cancer detection devices, because the HIS usually requires minutes to scan a target to capture the spatial and spectral data, and the volume of the HIS is usually high. The HIS or spectrometer can be used to find the characteristic spectrum for identifying the target, but the spectrum is difficult to directly implement in a multi-spectra system; the number of spectral bands is too large to practically allow implementation in a portable multispectral imager. Thus, a multispectral imager composed of a few determined band-pass filters is fundamentally important for the quick screening of oral cancer. 

Traditionally, an oral examination for oral cancer involves a visual inspection and palpation of oral lesions under illumination. If the clinician suspects there is a risk of abnormal tissue progressing to cancer tissue, an oral biopsy for histopathological analysis is necessary. It is a challenge for experienced clinicians to observe the superficial characteristics of oral cancer due to the subtle changes of epithelia in the cancer’s malignant progression. To enhance visualization for quick screening of oral cancer, several handheld assistant tools have been developed, such as VELscope [18], Identafi 3000 (DentalEZ Inc., Malvern PA, USA)) [19], and EVINCE (MMOptics, São Carlos, Brazil) [20]. These devices provide a light source from ultraviolet light-emitting diodes (LEDs) to excite the oral tissue and help an examiner observe the autofluorescence of oral tissue through a long-pass or band-pass optical filter. By observing the fluorescence loss, the development and progression of oral neoplasia can be artificially identified and scored using these devices. These devices have been used in clinician examinations and trials, showing their ability and efficacy for the screening of oral cancer [21,22,23,24,25,26,27,28,29,30,31]. Recently, Jeng et al. [32] developed a principle component analysis based method that combined a VELscope and Raman spectroscopy to improve the detection of oral cancer. Jeng et al. [32] further used linear discriminant analysis and quadratic discriminant analysis to increase differentiation between normal, premalignant, and malignant lesions based on the autofluorescence images acquired from the VELscope; the accuracy of the classifications was increased by 2% to 14% [33]. Huang et al. [34] created a two-channel autofluorescence detection that used 375 and 460 nm excitation light sources and 479 and 525 nm band-pass emission filters to detect oral cancer and precancerous lesions. The results revealed that autofluorescence had high sensitivity for detecting oral cancer. Cherry et al. [35] examined oral potentially malignant disorders (OMPDs) based on autofluorescence imaging and suggested that autofluorescence imaging had the potential to track OMPDs.

These beneficial tools mainly aim to enhance the visualization of autofluorescent loss in abnormal tissue. In this study, we developed a portable handheld multispectral imager to acquire the spectral image of the tumor and normal oral tissue in several bands and attempted to determine the effective spectral bands for identifying oral cancer based on several quantitatively computing methods. In a previous study, our team proposed a self-made portable LED-induced autofluorescence multispectral imager device for the screening of oral cancer [36]. This earlier device is mainly composed of LEDs, emission filters, and a CMOS imaging sensor, and was used to collect the spectral images of autofluorescence in normal and tumor tissues. The results of this study illustrated that the autofluorescence of the healthy and tumor tissues had significant variance in the blue intensity. However, how to determine appropriate band filters is a vital issue in portable devices, especially for quick screening of oral cancer. In the current study, the methodology for band selection of the LIAF multispectral imager was proposed and demonstrated using 436 sample points from 62 patients in ex vivo trials undertaken at China Medical University Hospital. Two light intensities and two wavelengths of the LEDs, 365 and 405 nm, were used as the excitation light sources; nine wavelengths of the spectral band, 470, 505, 525, 532, 550, 595, 632, 635, and 695 nm, were acquired. The spectral images were pre-processed by four image processing methods, including intensity, histogram, entropy, and fractional dimension. The threshold of the quantized value for screening the tumor points was optimized by calculating the area under the receiver operating characteristic curve (ROC). To find the effective spectral bands, a single-layer network learning process was used, and results were compared to a conventional rules-based process.

## 2. Materials and Methods

### 2.1. Instrument Composition and Spectral Characteristics

**Figure 1:**
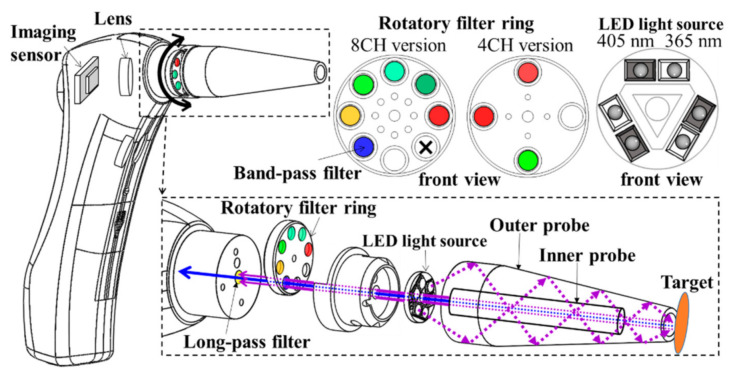
Mechanical drawing and explosion drawing of the LIAF multispectral imager.

The autofluorescence multispectral imager was equipped with excitation blue or purple LED sources, a long-pass filter suppressed from the excitation light sources, several band-pass filters, and a CMOS image sensor to capture the autofluorescence multispectral images from the reflection of specimens. The LIAF multispectral imager was minimized and implemented as a self-made, convenient-portable, and easy-handheld device, as shown in Figure 1. The device uses LEDs to induce the autofluorescence of target tissue and acquire the spectral images of the autofluorescence; the excitation LED light sources module was equipped with six excitation LEDs; the emission filters on the rotatory filter array passed the spectrum of the autofluorescence within a certain wavelength range of interest and rejected the spectrum without the wavelength range of interest; and the imaging system was composed of a color CMOS imaging sensor and lens capturing the fluorescent image induced from the tissues. The LEDs are placed on a Metal Core Printed Circuit Board (MCPCB), which can cool the heat produced by the LEDs. The current of LEDs is controlled using pulse width modulation techniques which are generated by the imaging module. The probe is in front of the holder blocking out the ambient light and fixing the object distance of the system. The filter ring contains band-pass filters arranged in a circle and can be rotated by the users’ fingers to change the filters. The target tissues are excited by the LEDs, which generate autofluorescence transmitting across the band-pass filter, long-pass filter, and the lens. The autofluorescence is captured by the imaging sensor afterward.

**Figure 2:**
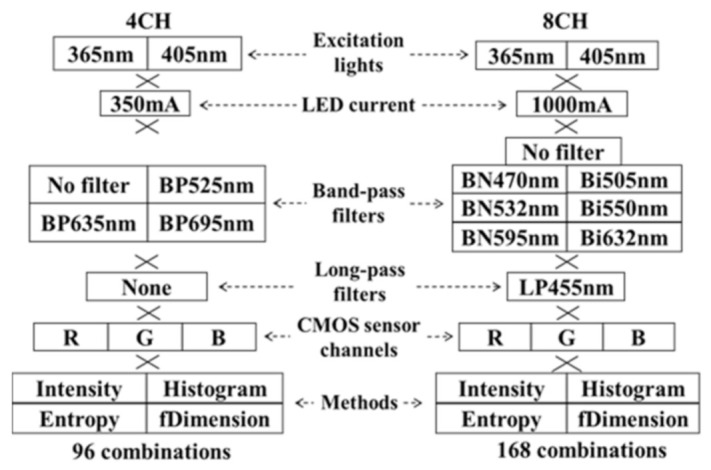
Composition of the 4CH LIAF multi-spectral imager and 8CH LIAF multi-spectral imager, including excitation lights, LED current, band-pass filters, a long-pass filter, and a CMOS sensor.

The LIAF multispectral imager was divided into four-channel (4CH) and eight-channel (8CH) versions based on the number of band-pass filters on the filter ring (Figure 2). Furthermore, the total current intensities of the LEDs were 350 mA in the 4CH version and 1000 mA in the 8CH version (Figure 2). The 4CH and the 8CH LIAF used the same excitation LEDs. The four channels in the rotary filter ring of the 4CH LIAF multispectral imager had three band-pass filters and one without filters. The center wavelengths of these filters were 525, 635, and 695 nm. The seven channels in the rotary filter ring of the 8CH LIAF multispectral imager had six band-pass filters and one without filters. The center wavelengths of these filters were 470, 505, 532, 550, 595, and 632 nm. To block the reflection of the excitation light entering the imaging system, a long-pass filter (LP455) was adopted in the 8CH LIAF multispectral imager (Figure 2); this filter can block the wavelength of light shorter than 455 nm and pass the wavelength of the light longer than 455 nm. Because the 4CH LIAF multispectral imager was the earlier version of the LIAF multispectral imager, the LP455 was not adopted in this version.

### 2.2. Patient History and Experimental Design

Patients who were referred to the Department of Otolaryngology-Head and Neck Surgery at China Medical University Hospital because of suspicious oral lesions or were waiting for head and neck surgery in the hospital ward were recruited to participate in the study. This study was reviewed and approved by the Institutional Review Board of China Medical University Hospital (CMUH102-REC1-069) [37], and the analysis was checked and approved by the Department of Biomedical Engineering, National Yang Ming Chiao Tung University, Taiwan. Written informed consent was obtained from each subject enrolled in the study. Patients in the region between 20 and 100 years of age were eligible to participate.

The patients involved in this study were divided into three experiments, as shown in Table 1. The first experiments (Exp_1) involved 17 patients using the 4CH LIAF multi-spectral imager without the correcting procedure. The second experiment (Exp_2) involved 19 patients using the 4CH LIAF multi-spectral imager with the correcting procedure. The third experiment (Exp_3) involved 26 patients using the 8CH LIAF multi-spectral imager with the correcting procedure. The anatomical specimens were collected from the surgical resection of the different patients, except for two anatomical specimens collected from one patient in Exp_3. Thus, a total of 28 specimens were involved in Exp_3.

**Figure 3:**
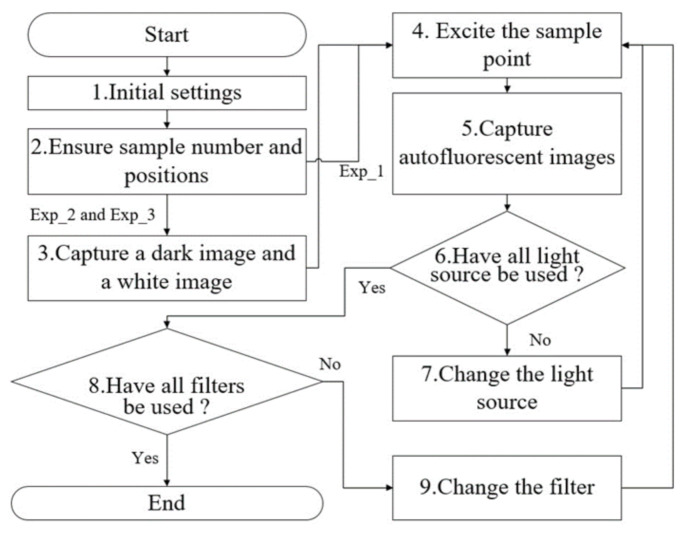
Flowchart of the trials.

**Figure 4:**
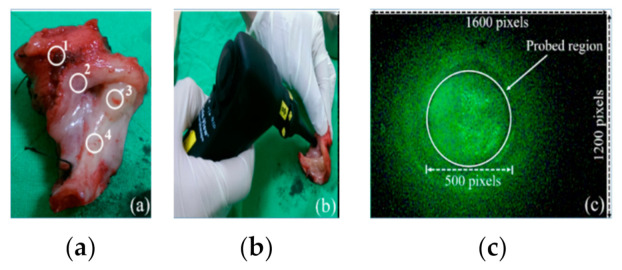
(**a**)Tumor regions and normal regions, which are captured are marked as a white circle. (**b**) LIAF captures the tumor or normal points. (**c**) Captured image with a circle indicating the opening scope of the probe.

The experiment procedure is shown in Figure 3. After the instruments were set and the surgeon completing the surgical operation, the surgeon immediately selected several points of the tumor region and healthy region on the anatomical specimen (Figure 4a). The third step was to capture a dark image and a white image of white balance and diffuse reflectance targets. The dark image and white image were used to perform a dark calibration, and a white calibration for reducing the intensity offset produced from the imaging sensor and reducing the impact of the various flux in the light source. The surgeon aimed the LIAF multi-spectral imager at each selected point and pressed the trigger of the LIAF multi-spectral imager to start the capturing procedure (Figure 4b). The capturing procedure comprised steps 4 to 9. The third step was to capture the dark image without emitting light. The fourth step was to excite the point, and the fifth step was to capture the autofluorescence image of the point (Figure 4c). The third step to the fifth step was performed again after the light source was changed automatically. After the capturing procedure was completed, the indicator of the LIAF multi-spectral imager was illuminated. The surgeon changed the band-pass filter by rotating the rotary filter ring and started the next capturing procedure. The surgeon repeated the above operation until the active channels of the rotary filter ring were all used. Sequentially, the surgeon repeated the third to ninth steps for all points. As shown in Table 2, a total of 53 healthy points and 53 tumor points were collected in Exp_1; a total of 54 healthy points and 54 tumor points were collected in Exp_2; and a total of 111 normal points and 111 tumor points were collected in Exp_3.

### 2.3. Data Collection and Analysis

The spectral images of the specimens captured from the selected points of tumor tissue and normal tissue were used in the analysis. The pixels of one image used for analysis were only in the probe region (Figure 4c). Because the SOI-268 is a color CMOS imaging sensor, one image contains red (R), green (G), and blue (B) gray-level images. Three gray-level images were used as an independent data set. The first step was to analyze the images of the points depending on four methods, including intensity, histogram, fractal dimensions, and entropy. The first method calculated the summation of the gray level of the pixels in the probe region (ROI) of one spectral image. The summation, *I*, is expressed as:

(1) $$I=\sum_{x,y\in ROI}f\left(x,y\right),$$

where *f* is the gray level of the pixel at coordinate (*x*,*y*) that is in the ROI. The second method was to find the highest number *S* of the intensity level in a spectral image:

(2) $$S=\max\left\{n_{k}\right\},$$

where *n_k_* is the number of pixels in *f* with intensity *r_k_* that denotes the intensities of an *L*-level spectral image; *k* is from 0 to *L*-1. In information theory, entropy measures uncertainty in a set of random variables. The third method was to calculate the entropy *H*, expressed as:

(3) $$H=-\sum_{i=0}^{n}P\left(r_{k}\right)\times \log_{2}P\left(r_{k}\right),$$

where *P*(*r_i_*) is the proportion of the gray level *r_k_* in a spectral image. The fourth method uses the concept of fractal dimension. The fractal dimension is an index that characterizes the complexity of a pattern. The morphological shape of the tumor tissue could be chaotic because of the random proliferation of the tissue. Thus, the fractal dimension may be useful in oral cancer detection. The first step of the fourth method was to binarize the images using Otsu’s method. Otsu’s method finds the optimum threshold of $r_{k}$ when the maximizing inter-class variance *σ*($r_{k}$) is found. $\sigma^{2}\left(r_{k}\right)$ is expressed as:

(4) $$\sigma^{2}\left(r_{k}\right)=\sum_{k=0}^{t-1}P\left(r_{k}\right)\times \sum_{k=t}^{L-1}P\left(r_{k}\right)\times {\left[\frac{\sum_{k=0}^{t-1}r_{k}\times P\left(r_{k}\right)}{\sum_{k=0}^{t-1}P\left(r_{k}\right)}-\frac{\sum_{k=t-1}^{L-1}r_{k}\times P\left(r_{k}\right)}{\sum_{k=t-1}^{L-1}P\left(r_{k}\right)}\right]}^{2},$$

where *k* ranges from 0 to *L*. The optimum threshold *t* was used to transform a gray-level image *f* into a binary image *f_b_*. The transfer is expressed as:

(5) $$f_{b}\left(x,y\right)=\left\{\begin{array}{c} \;1,\;f\left(x,y\right)\geq \;t\\ 0,\;f\left(x,y\right)<t \end{array}\right..$$

A kernel of size 2 by 2 pixels moved over the binary image *f_b_* and computed the sum of the product at each location. *f_c_* is the result of the spatial correlation and is expressed as:

(6) $$f_{c}\left(i,j\right)=\left\{\begin{array}{c} \;1,\;\;\sum_{x=i,y=j}^{i+1,j+1}f_{b}\left(x,y\right)\geq 1\;\\ 0,\;\;\sum_{x=i,y=j}^{i+1,j+1}f_{b}\left(x,y\right)<1 \end{array}\right..$$

After the spatial correlation, the summation of *f_c_* was calculated as follows:

(7) $$D=\sum_{i=0,j=0}^{M-2,N-2}f_{c}\left(i,j\right),$$

where *M* and *N* are the dimensions of *f_c_*.

The red, green, and blue filters were regarded as three spectral bands and correlated with the emission filters to become new spectral bands; thus, a total of 30 spectral bands are listed in Table 3. In addition, a total of two excitation light sources and four image processing methods were used; thus, a total of 96 and 168 combinations were regarded as different classifiers in Exp_2 and Exp_3, respectively (Figure 2). The data of each combination were divided into two groups, A and B, for the cross-validation, as shown in Table 2. One group was the training set, and the other group was a validation set. The training set was used to optimize the threshold for distinguishing the normal from the tumor points. The optimized threshold was tested in a validation set to derive the sensitivity, specificity, and accuracy of the classifier. 

**Figure 5:**
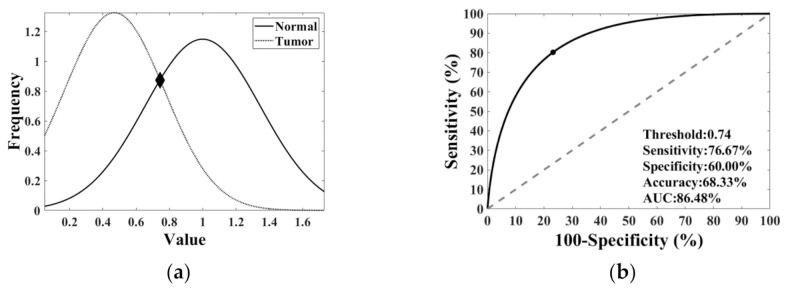
(**a**) Gaussian distribution of normal and tumor data. The Gaussian distributions of normal and tumor data are drawn as a solid line and dotted line, respectively. The crossing point marked as “◆” represents the optimized threshold. (**b**) The ROC curve of normal and tumor data in one classifier. The crossing point marked as “•” represents the optimized threshold.

The threshold was the cross point of two Gaussian distributions of normal and tumor data. The normal and tumor data in which the *p*-value of the Kolmogorov–Smirnov (KS) tests [32] exceeded 0.05 were assumed to have a Gaussian distribution. The Gaussian distribution of the normal and tumor data was determined by their mean and the standard deviation. These distributions were used to find the optimized threshold and calculate the receiver operating characteristic curve (ROC). One threshold determines one sensitivity standing for a fraction of true positives to all tumor points and one specificity standing for a fraction of true negatives to all normal points. The accuracy standing for a fraction of true positive and true negative to all points was also determined (Table 4). The ROC curve is plotted with the sensitivities and one minus specificity of the various threshold (Figure 5). The optimal threshold marked as ‘‘filled circle’’ was determined by the highest accuracy, sensitivity, and specificity on the ROC (Figure 5). The area under the ROC curve (AUC) was used to evaluate the performance of the classifiers [38,39,40,41].

## 3. Results

### 3.1. LAIF Spectral Imaging with and without Filters

**Figure 6:**
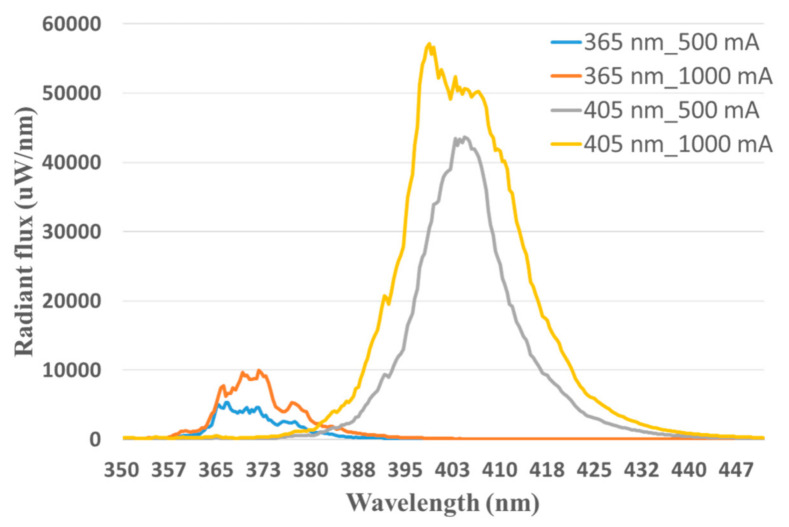
Spectral radiant flux of the excitation LEDs.

**Figure 7:**
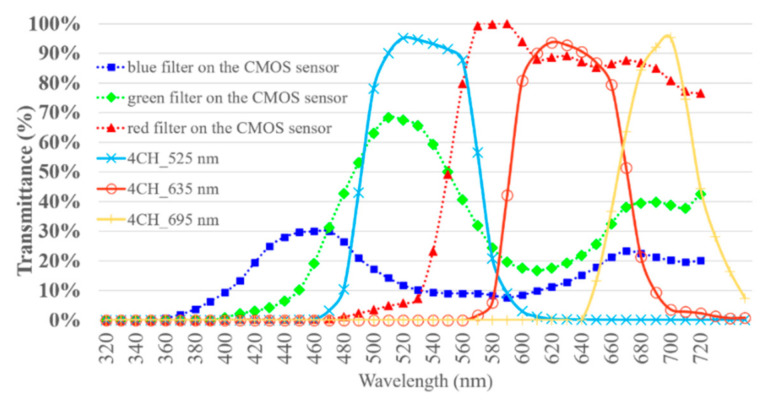
Spectral transmittance of the emission filters and RGB color filters of the 4CH LIAF multi-spectral imager [43,44,45,46].

**Figure 8:**
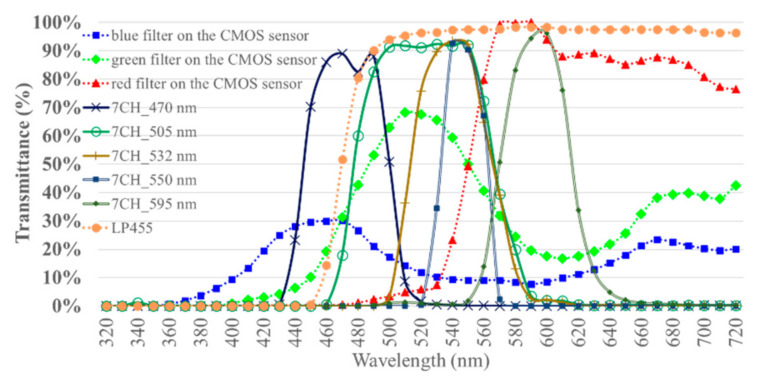
Spectral transmittance of the emission filters, long-pass filter, and RGB color filters of the 8CH LIAF multi-spectral imager [43,47,48,49,50,51,52,53].

The spectrum, radiant flux, and luminous flux of the excitation LEDs were measured using a SMS-500 spectrometer in conjunction with an integrating sphere. The measurement was compliant with CIE 127:2007 [42]. First, the spectral calibration and absolute luminous flux calibration of the measurement system were implemented. Then, the excitation LEDs were installed in the integrating sphere. The spectrum, radiant flux, and luminous flux of the excitation LEDs driving the forward currents of 500 and 1000 mA were each measured ten times. The ten records of each condition were averaged. The spectral radiant flux of the excitation light sources is shown in Figure 6. The total radiant flux of the 365 nm LEDs driving the forward current of 500 and 1000 mA was 76,613 and 141,663 µW. The total radiant flux of the 405 nm LEDs driving the forward current of 500 and 1000 mA was 782,890 and 1,267,606 µW. The peak wavelength of the 365 nm LEDs and 405 nm LEDs was 365 and 401 nm. The full width at half maximum (FWHM) of the 365 nm LEDs ranged from 364.26 to 375.64 nm. The FWHM of the 405 nm LEDs ranged from 395.55 to 411.95 nm. The designed peak wavelength of the 405 nm LEDs differed from the measured peak wavelength but was still in the measured FWHM. The dominant wavelength of the 365 nm LED was 462 nm. The CIE *x* and *y* of the 365 nm LEDs were 0.2182 and 0.1607, respectively, which approaches the purple–blue color. The dominant wavelength of the 405 nm LED was 431 nm. The CIE *x* and *y* of the 405 nm LEDs were 0.1742 and 0.0188, respectively, which approaches the blue color. The relative spectral intensity of the excitation light sources is shown in Figure 6. The 8CH LIAF and the 4CH LIAF multi-spectral imager used the SOI-268 CMOS sensor. The transmittance of the red, green, and blue filters correlated with the spectral response of the sensor are marked as dotted lines and the spectral transmittance of the emission filters used in the 4CH version are marked as solid lines in Figure 7. The spectral transmittance of the emission filters used in the 8CH version is marked as solid lines in Figure 8; the spectral transmittance of long-pass filter LP455 is plotted in Figure 8 as dotted lines with cross marks. The peak wavelength and the corresponding transmittance of the red, green, and blue filter correlated with and without each emission filter are shown in Table 3.

### 3.2. Band Selection for Oral-Cancer Diagnosis

**Figure 9:**
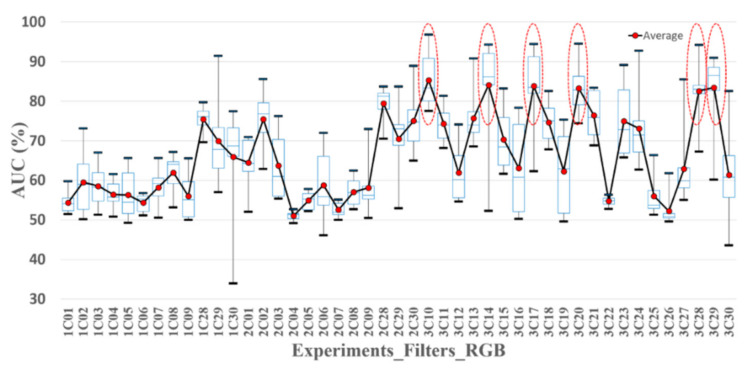
The AUC statistic of the classifiers with 30 spectral bands in Exp_1, Exp_2, and Exp_3. The data in each classifier was used to calculate the AUC (A + B). The solid red points are the average AUCs. The highest and lowest black bold dash lines are the maximum and minimum AUC. The highest and the lowest blue dash lines are third and first quartile AUC. The mid-blue dashed lines are the median AUC.

**Figure 10:**
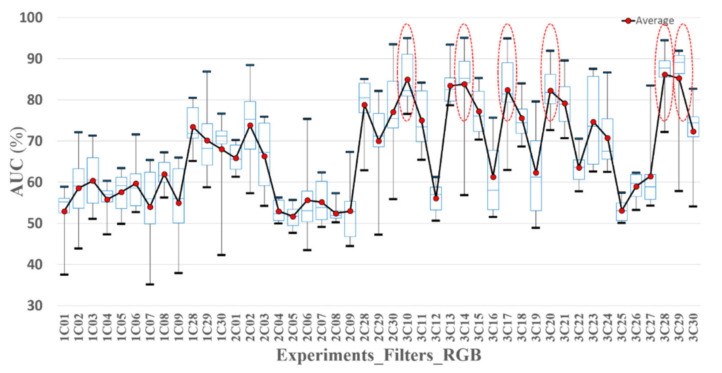
The AUC statistic of the classifiers with 30 spectral bands in Exp_1, Exp_2, and Exp_3. The data in group A was used to calculate the AUC. The solid red points are the average AUCs. The highest and lowest black bold dash lines are the maximum and minimum AUC. The highest and the lowest blue dash lines are third and first quartile AUC. The mid-blue dashed lines are the median AUC.

**Figure 11:**
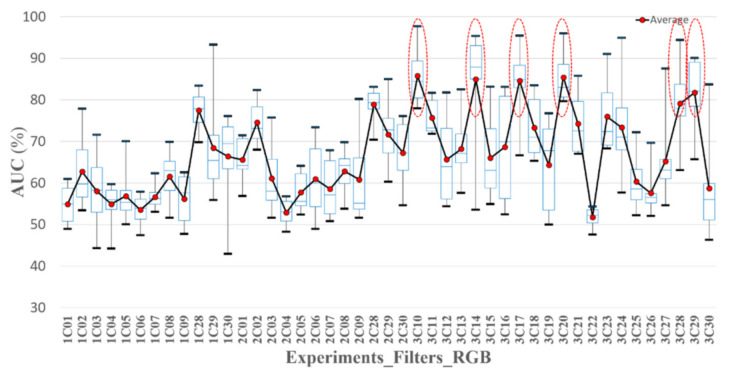
Statistic AUC of the classifiers with 30 spectral bands in Exp_1, Exp_2, and Exp_3. The data in group B was used to calculate the AUC. The solid red points are the average AUCs. The highest and lowest black bold dash lines are the maximum and minimum AUC. The highest and the lowest blue dash lines are third and first quartile AUC. The mid-blue dashed lines are the median AUC.

In this study, we attempted to identify effective spectral bands for the screening of oral cancer. The selection of the spectral bands was based on the AUC of the classifiers tested in a training set and the sensitivity, specificity, and accuracy of the classifiers tested in a validation set. A total of thirty spectral bands were collected from the R, G, and B filter, correlated with and without the nine emission filters. Each spectral band was included in eight classifiers associated with two excitations and four image processing methods. The data of each classifier was divided into two groups, A and B. One group was a training set, and the other one was a validation set. For evaluating the performance of the classifiers, the AUC and the optimized threshold of the training set were calculated. The threshold was used to test the validation set to calculate the sensitivity, specificity, and accuracy. The minimum, first quartile, second quartile, third quartile, average, and maximum AUC of the group A, B, and A + B in eight classifiers of 30 spectral bands were calculated and are depicted in Figure 9, Figure 10 and Figure 11, respectively. The average AUC of 3C10 (470 nm_B), 3C14 (505 nm_G), 3C17 (532 nm_G), 3C20 (550 nm_G), 3C28 (B), and 3C29 (G) was higher than the others.

#### 3.2.1. Rules-Based Band Selection

**Figure 12:**
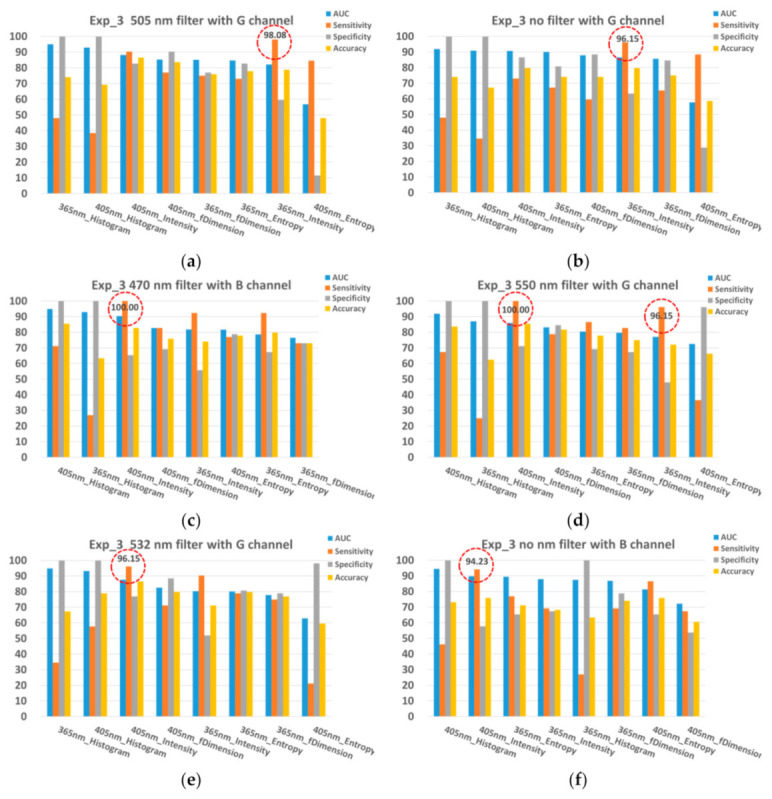
(**a**) Sensitivity, specificity, and accuracy of the classifiers using the 505 nm emission filters correlated with the green filter. (**b**) Sensitivity, specificity, and accuracy of the classifiers using the green filter. (**c**) Sensitivity, specificity, and accuracy of the classifiers using the 470 nm emission filters correlated with the blue filter. (**d**) Sensitivity, specificity, and accuracy of the classifiers using the 550 nm emission filters correlated with the green filter. (**e**) Sensitivity, specificity, and accuracy of the classifiers using the 532 nm emission filters correlated with the green filter. (**f**) Sensitivity, specificity, and accuracy of the classifiers using the blue filter. Each spectral band which is the emission filter correlated with the red, green, and blue filters has eight classifiers according to the excitation LEDs and the methods. The red dotted circles indicate the classifiers with the highest sensitivity (over 94%).

For further band selection, the top four AUCs of each spectral band were only considered and taken into the average for ranking; that is, the four worst AUCs of each spectral band were not taken into account. The top six average AUC values of the spectral bands are shown in Table 5. The spectral bands, namely 3C14, 3C10, 3C17, 3C29, 3C20, and 3C28, ranked in the top six AUCs of the groups A, B, and A + B. The results illustrate that the blue and green filter correlated with and without four emission filters of center wavelength 470, 505, 532, and 550 nm exhibited the best performance for the screening of oral cancer. Combined with selecting the spectral bands, the effective excitation light source and image processing methods were further selected. The aim of rapid screening of oral cancer is to efficiently identify suspicious oral mucosa tissues in the early stage of oral cancer; the high sensitivity of the screening result is preferable relative to its high specificity; thus, the criteria for selecting the effective excitation light source and the imaging processing methods were based on the sensitivity of the testing results. The sensitivity, specificity, and accuracy of the validating results in 3C14, 3C10, 3C17, 3C29, 3C20, and 3C28 are shown in Figure 12. The classifiers with the highest sensitivity (over 94%) in the validating results were selected and are marked as red dotted circles in Figure 12.

#### 3.2.2. AI-Based Band Selection

**Figure 13:**
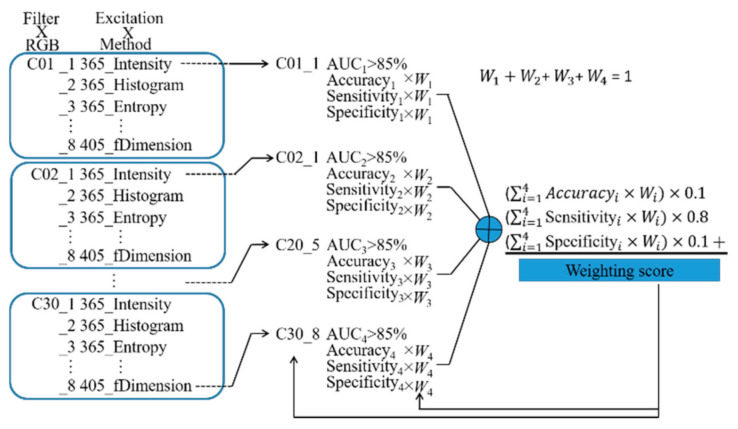
AI-based band selection method flow chart. Four spectral bands are selected from C01 to C30 in the first stage. The classifier whose AUC is greater than 85% is selected from each selected spectral band. A total of four classifiers are selected in the second stage. The accuracy, sensitivity, and specificity of four selected spectral bands are multiplied by the corresponding weighting score *W* and summed. The summed accuracy is multiplied by 0.1, the summed sensitivity is multiplied by 0.8, and the summed specificity is multiplied by 0.1. The weighting sore is the summation of the three multiplied values. The method adjusts the weightings to find the maximum weighting score for each selection, and the weighting score of each selection is calculated and compared to find the best filter groups.

An artificial intelligence (AI) method was used to select the spectral bands. The method is illustrated in Figure 13. In iteration I, the spectral bands in which the average AUC was over 85% were selected. In iteration II, the accuracy, sensitivity, and specificity of each classifier in these selected spectral bands were used to calculate the weighting core. The method optimized the weighting *W* to find the maximum weighting score of the classifiers. The classifier which had the highest weighting score in one of the selected spectral bands determined the effective excitation light sources and imaging processing methods in conjunction with this spectral bands. For the screening of oral cancer, high sensitivity is more important than high specificity; thus, the sensitivity has the highest weighting value (0.8) compared to specificity and the accuracy. The selected spectral bands of the AI-based method in the data without grouping (A + B), group A, and group B are shown in Table 6. These four spectral bands were also selected using the rules-based method, namely 3C14, 3C17, 3C20, 3C28.

## 4. Discussion and Conclusions

In this study, a method of the screening of oral cancer was used to observe the autofluorescence of the tissue after the tissue was excited by LEDs. The autofluorescence was filtered by the different RGB filters with or without emission filters and recorded by the imaging sensor. In the experiments, we adopted the red, green, and blue filters in the SOI268 with or without the emission filters, which have center wavelengths of 525, 635, 695, 470, 505, 532, 550, 595, and 632 nm. The most important purpose of this paper was to select the effective filters for the rapid screening of oral cancer. In rules-based band selection, the spectral bands which identically ranked among the top six average AUCs of the classifiers tested in the data group A, B, and the data without grouping (A + B), were C14, C10, C17, C29, C20, and C28; the average of the top four AUCs in the test of eight classifiers in these spectral bands was greater than 85% (Table 5). The AI-based method selected the four spectral bands, which were the same as the those for the selection of the rules-based method. The blue and the green filter of the SOI268 CMOS imaging sensor showed good performance with or without the emission filters. Furthermore, the emission filters with center wavelengths of 550, 532, 470, and 505 nm showed better performance than other filters. 

The goal of the LIAF multi-spectral imager is to allow rapid screening at the early stage of oral cancer. For quick screening at an earlier stage, high sensitivity of testing results is more important than high specificity. Therefore, the selection of the excitation LED wavelength and the computing methods aimed to find the classifiers which had the highest sensitivity. Each spectral band was involved in eight classifiers that used two excitation light sources and four quantitative computing methods. In the rule-based method, if the sensitivity of the classifier is higher than 90%, the classifier is adopted. According to the results (Figure 12), a total of six classifiers were selected, including a 365 nm excitation LED with the intensity method in C14, a 405 nm excitation LED with the intensity method in C10, a 405 nm excitation LED with the intensity method in C17, a 365 nm excitation LED with the intensity method in C29, a 405 nm excitation LED with the intensity method in C20, and a 405 nm excitation LED with the intensity method in C28. In the AI-based method, the final six combinations were selected, including a 405 nm LED with the intensity method for C14, a 405 nm LED with the intensity method for C10, a 405 nm LED with the intensity method for C17, a 365 nm LED with the intensity method for C29, a 405 LED with the intensity method for C20, and a 405 nm LED with the intensity method for C28. These classifiers in the rules-based and AI-based methods were identically adopted. The sensitivity of these six classifiers was larger than 90%. The average of the sensitivities to these six classifiers was 96.15%; the average of the specificities was 69.55%; and the average of the accuracies was 82.85%. For the investigated application, these six classifiers could be implemented in a LIAF multispectral imager for the quick screening of oral cancer.

The methodology of the AI-based band selection used the area under the ROC curve (AUC) to evaluate the performance of all classifiers. The advantage of the ROC and AUC is that the performance of the combinations does not depend on the threshold. Furthermore, the AI-based method was successfully verified by the ruled-based method. The advantage of the AI-based method is that the method can adjust the weighting to rapidly identify the best four combinations consisting of four bands. The weightings can decide the means of detection based on the four selected classifiers with four spectral bands. The rule-based method is based on the AUC ranks of the spectral bands in the data without grouping (A + B), group A, and group B to select the final four spectral bands. However, the rules-based method is unable to determine the means of detection based on the six selected classifiers with four spectral bands and cannot adjust itself to improve the testing results. The construction of the rule-based method is based on considerable background knowledge and is time-consuming. Therefore, the rules-based method can be replaced by the AI-based method. In the future, the AI-based methodology of the band selection and the combination selection can be applied to other cancer detection for quick screening.

Pioneer studies used an optical spectroscope to investigate the autofluorescent spectrum of the normal and abnormal oral tissue, and found that the autofluorescence intensity of the abnormal tissue decreased in blue and green spectral regions compared to that of normal oral tissue [6,7,8,9,10,11,12,13,14,15,16]. The selected spectral bands in this study are consistent in the blue and green regions. However, the specific spectral bands did not encompass or were not equal to the peak wavelength of autofluorescent for identifying oral cancer in previous studies. The peak wavelength of excitation light sources was not consistent in all studies because the excitation band of the fluorophores is broad [6,53]. The spectroscope might not be suitable to quickly screen and demarcate oral cancer in the whole oral cavity due to the narrow view and single measured point of most spectroscopes [53]. Several handheld assistant tools, such as VELscope, Identafi, and EVINCE, have been developed to enhance the visualization of oral lesions based on observing the loss of green or blue autofluorescence in abnormal tissue [18,19,20]. In most studies in which these tools have been successfully used in clinical trials, examiners or surgeons identified the oral lesion using the tool or the image captured with the tool [21,22,23,24,25,26,27,28,29,30,31,54,55,56,57,58,59,60,61,62,63,64,65,66,67,68,69,70]. In this study, we developed a portable handheld LED-induced autofluorescence multispectral imager that might be more suitable for quick screening of oral cancer compared to optical spectroscopy or HIS. For this reason, 550, 532, 470, and 505 nm emission filters used in conjunction with 365 and 405 nm excitation LEDs were selected to enhance the differentiation between tumor tissue and normal tissue. These filters were implemented in a multi-spectral imager, and the computing methods used were able to provide a quantitative value for identifying oral cancer without requiring the opinion of experts.

## Figures and Tables

**Table 1:** Characteristics of the subjects in three experiments.

Experiment	Exp_1	Exp_2	Exp_3
Version of the imaging system	4CH	4CH	4CH
Dark image correction	no	yes	yes
Patient	17	19	26
Male: Female	17/00	16/03	23/03
Specimen (sample points)	17 (106)	19 (108)	28 (222)
Age	54 ± 14	58 ± 12	58 ± 11

**Table 2:** Information of the specimens and the sample points in three experiments.

Experiments	Total Sample Points			Testing Group					
				Group A			Group B		
	Specimens	Normal Points	Tumor Points	Specimens	Normal Points	Tumor Points	Specimens	Normal Points	Tumor Points
Exp_1	17	53	53	9	27	27	8	26	26
Exp_2	19	54	54	10	28	28	9	26	26
Exp_3	30	111	111	15	51	51	15	60	60

**Table 3:** Spectral transmittances of the R, G, and B filters correlated with and without the emission filters.

No.	Spectral Bands	Band Wavelength (nm)	The Central Wavelength of the Spectral Transmittance	No.	Spectral Bands	Band Wavelength (nm)	The Central Wavelength of the Spectral Transmittance
C01	525 nm_B	510 ± 30.0	0.24	C16	532 nm_B	520 ± 20.0	0.16
C02	525 nm_G	540 ± 30.0	0.64	C17	532 nm_G	540 ± 25.0	0.64
C03	525 nm_R	570 ± 15.0	0.13	C18	532 nm_R	570 ± 10.0	0.09
C04	635 nm_B	660 ± 40.0	0.10	C19	550 nm_B	540 ± 15.0	0.13
C05	635 nm_G	600 ± 42.5	0.26	C20	550 nm_G	540 ± 15.0	0.63
C06	635 nm_R	620 ± 42.5	0.94	C21	550 nm_R	550 ± 15.0	0.05
C07	695 nm_B	700 ± 25.0	0.22	C22	595 nm_B	600 ± 20.0	0.09
C08	695 nm_G	700 ± 22.5	0.36	C23	595 nm_G	580 ± 250	0.42
C09	695 nm_R	700 ± 27.5	0.84	C24	595 nm_R	600 ± 17.5	0.95
C10	470 nm_B	490 ± 25.0	0.26	C25	632 nm_B	640 ± 15.0	0.09
C11	470 nm_G	490 ± 15.0	0.17	C26	632 nm_G	640 ± 17.5	0.16
C12	470 nm_R	750 ± 10.0	0.03	C27	632 nm_R	630 ± 17.5	0.82
C13	505 nm_B	500 ± 30.0	0.28	C28	Blue	500 ±50.0	0.30
C14	505 nm_G	540 ± 35.0	0.63	C29	Green	540 ±45.0	0.68
C15	505 nm_R	580 ± 15.0	0.10	C30	Red	620 -40.0	1

**Table 4:** Definition of the sensitivity, specificity, and accuracy.

		True Condition	
		Condition Positive	Condition Negative
Predicted condition	Predicted Positive	True-Positive (TP)	False-Positive (FP)
	Predicted Negative	False-Negative (FN)	True-Negative (TN)
		Sensitivity = TP/(TP + TN)	Specificity = TN/(FP + FN)
		Accuracy = (TP + TN)/(TP + TN+ FP + FN)	

**Table 5:** Average AUCs (%) of group A + B, A, and B.

A + B		A		B	
No.	AUC	No.	AUC	No.	AUC
**3C14**	91.52	**3C29**	90.93	**3C14**	92.76
3C10	90.74	3C28	90.39	3C10	90.92
**3C17**	90.62	3C14	90.36	**3C17**	90.19
3C29	89.08	**3C10**	90.27	**3C20**	90.04
**3C20**	88.49	**3C17**	89.58	**3C29**	87.93
**3C28**	86.63	**3C20**	87.00	3C28	85.34

**Table 6:** Results of AI-based band selection in three data groups.

	No.	Excitation	Method	Weighting (*W*)
A + B	3C20	405 nm	Intensity	0.7
	3C14	405 nm	Intensity	0.1
	3C17	405 nm	Intensity	0.1
	3C28	405 nm	Intensity	0.1
A	3C20	405 nm	Intensity	0.7
	3C10	405 nm	Intensity	0.1
	3C17	405 nm	Intensity	0.1
	3C29	365 nm	Intensity	0.1
B	3C14	405 nm	fractal dimension	0.7
	3C17	405 nm	Intensity	0.1
	3C20	405 nm	Intensity	0.1
	3C29	365 nm	Histogram	0.1

## Data Availability

The data presented in this study are available on request from the corresponding author. The data are not publicly available due to their containing information that could compromise the privacy of research participants and the IRB statement (CMUH102-REC1-069).
